# Multiplex PCR followed by restriction length polymorphism analysis for the subtyping of bovine herpesvirus 5 isolates

**DOI:** 10.1186/1746-6148-9-111

**Published:** 2013-06-04

**Authors:** Silvina Soledad Maidana, Cintia Débora Morano, Daniela Cianfrini, Fabrício Souza Campos, Paulo Michel Roehe, Bianca Siedler, Gabriel De Stefano, Axel Mauroy, Etienne Thiry, Sonia Alejandra Romera

**Affiliations:** 1 Instituto de Virología, Centro de Investigaciones en Ciencias Veterinarias y, Agronómicas (CICVyA), Instituto de tecnología Agropecuaria (INTA), N. Repetto, y Los Reseros S/N, CC25, (B1712WAA), Castelar, Buenos Aires, Argentina; 2 Consejo Nacional de Investigaciones, Científicas y Tecnológicas (CONICET), Rivadavia 1917, (C1033AAJ), Ciudad Autónoma de Buenos Aires, Argentina; 3 Inmunología, Universidad del Salvador, Champagnat 1599-Ruta Panamericana-Km 54.5 Pilar, -B1630AHU-Provincia de Buenos Aires, Argentina; 4 Tecnovax SA, Luis Viale 2835, 1416, Ciudad Autónoma de Buenos Aires, Argentina; 5 Virology Laboratory, Department of Microbiology, Immunology and Parasitology, Institute of Basic Health Sciences, Federal University of Rio Grande do Sul (UFRGS), Av. Sarmento Leite 500,Porto Alegre 90050-170, Rio Grande do Sul (RS), Brazil; 6 Laboratório de Bioprocessos, Universidade Federal de Pelotas, Rua Gomes Carneiro, 1, 96010-610, Pelotas, Brasil; 7 Veterinary Virology and Animal Viral Diseases, Department of Infectious and Parasitic Diseases Faculty of Veterinary Medicine, University of Liège, Liège, Belgium

## Abstract

**Background:**

Several types and subtypes of bovine herpesviruses 1 and 5 (BoHV-1 and BoHV-5) have been associated to different clinical conditions of cattle, making type/subtype differentiation essential to understand the pathogenesis and epidemiology of BoHV infections. BoHV-5 subtyping is currently carried out by *BstE*II restriction enzyme analysis (REA) of the complete virus genome. This method allowed the description of three subtypes, one of which is the most widespread while the remaining two have so far only been found in South America. The present work describes a multiplex PCR followed by REA for BoHV-5 subtyping.

**Results:**

The method consists in the simultaneous amplification of glycoprotein B and UL54 gene fragments of 534 and 669 base pairs (bp), respectively, *BstE*II digestion of amplicons, separation of products in 1% agarose gels, and analysis of fragment length polymorphims. The multiplex PCR detected up to 227 BoHV-5 genome copies and 9.2 × 10^5^ BoHV-5 genome copies when DNA was extracted from purified virus or infected tissue homogenates, respectively. The applicability of multiplex PCR-REA was demonstrated on 3 BoHV-5 reference strains. In addition, subtyping of two new isolates and seventeen previously reported ones (17 BHV-5a and 2 BHV-5b) by this method gave coincident results with those obtained with the classic *BstE*II REA assay.

**Conclusions:**

Multiplex PCR-REA provides a new tool for the fast and simple diagnosis and subtyping of BoHV-5.

## Background

Bovine herpesvirus 5 (BoHV-5) is an alphaherpesvirus responsible for meningoencephalitis in young cattle, and is antigenically and genetically closely related to bovine herpesvirus 1 (BoHV-1) [1]. The origin and geographic distribution of BoHV-5 infections are largely unknown, mainly due to serological cross-reactivity with BoHV-1 [2]. Sporadic cases of meningoencephalitis by BoHV-5 have been reported in Australia [3], USA [4], Italy [5] and Hungary [6]. In contrast, BoHV-5 infection and disease appear to be more frequent in Argentina and Brazil, where numerous outbreaks were described in the last decades [7-11]. The rare occurrence of BoHV-5 neurological disease in areas where BoHV-1 infection is endemic may be explained by cross-protection induced by natural infection or vaccination [12-14].

BoHV-5 infection induces either a subclinical infection or disease of moderate severity in adult cattle [15] and lethal encephalitis in young animals [6,7,16].

Virological assays are very accurate tools to specifically diagnose BoHV-5 infections. Virus isolation in cell culture can be performed from fresh or frozen nasal secretions, semen or post mortem samples [1]. However, in light of recent reports of bovine herpesviruses isolated from different samples, and responsible for both symptomatic and asymptomatic infections [11,17,18], classical diagnostic methods are not sufficient for a fast and easy identification and subtyping of the infectious virus.

Several assays are available to differentiate BoHV-5 from BoHV-1, including immunoassays using monoclonal antibodies [19-21], PCR followed by REA [22], nested PCR [13], multiplex PCR [23-25], random amplified polymorphic DNA (RAPD) [26], and multiple PCR sequencing assays [27]. Moreover, *BstE*II restriction enzyme analysis (REA) of the complete virus genome can differentiate between BoHV-5 subtypes; but the technique is laborious and needs substantial amounts and quality of viral DNA [28,29]. In addition, a recently described UL27PCR-REA [11] assay has been shown to differentiate between BoHV-5 subtypes a and b but it cannot differentiate the subtype c.

We here describe a molecular technique that allows the detection and differentiation of all BoHV-5 subtypes. We also report the identification and characterization of two new BoHV-5 isolates from the Argentinean Provinces of Buenos Aires and Chaco. One of them corresponds to a symptomatic case obtained during an outbreak of neurological disease in a cattle herd, while the other corresponds to a non symptomatic case isolated from a bovine semen sample.

This development could be epidemiologically relevant in areas where BoHV-5 infection is endemic, and provides a new tool for the fast diagnosis and subtyping of BoHV-5.

## Results

### Differential PCR for BoHV1 and BoHV5

The new virus isolates 674 and 2010 were tested by multiplex PCR [24] and shown to be BoHV-5.

### REA

**Figure 1:**
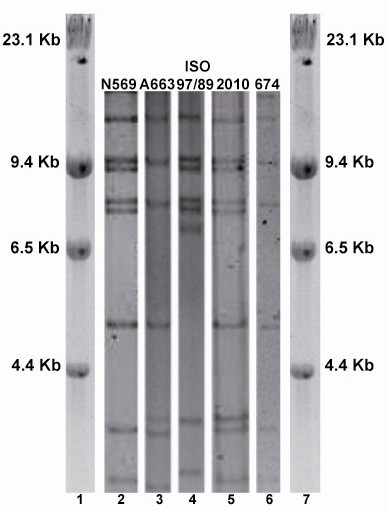
**Subtyping of new BoHV-5 isolates from Argentina. ***BstE*II restriction endonuclease profiles of isolates 674 and 2010 (lanes 5 and 6) and N569, A663 and ISO97/89 reference strains (lanes 2, 3 and 4), M: DNA size marker (lambda Hind III, Invitrogen).

One of the new field isolates (2010) showed a *BstE*II REA pattern similar to reference strain N569, which is the BoHV-5a prototype, and the other (674) showed a pattern similar to reference strain A663, the BoHV-5b prototype (Figure 1). A double 3 kb band was observed in the first case, but it fell outside of the area used for subtype classification.

### Determination of a differential restriction site for a and c subtypes

**Figure 2:**
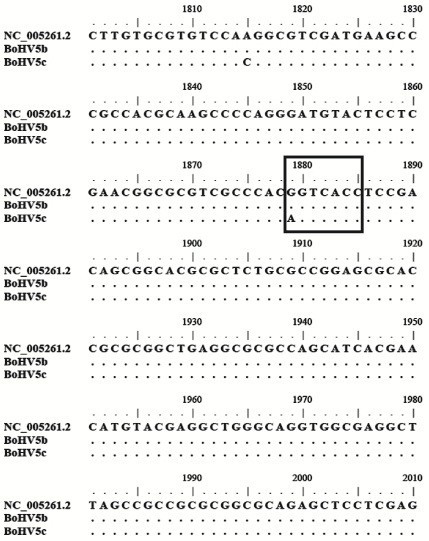
**Multiple nucleotide alignment of UL54 sequences from BoHV-5a (Genbank accession numbers NC_005261.2), BoHV-5b (A663) and BoHV-5c (ISO97/89).** Dots indicate conserved nucleotides. *BstE*II restriction site has been boxed.

Fragments (500 bp) of the UL54 gene from BoHV-5 subtypes a (N569), b (A663 reference strain) and c (ISO97/89) were sequenced. Sequence alignment revealed a point mutation (G to A) in the *BstE*II restriction site for subtype c (1879 nt position, NC_005261.2), as compared to subtype a, responsible for the variation in the REA pattern (Figure 2). This point mutation did not result in an amino acid change.

### Multiplex UL27/UL54 PCR and *BstE*II restriction analysis

**Figure 3:**
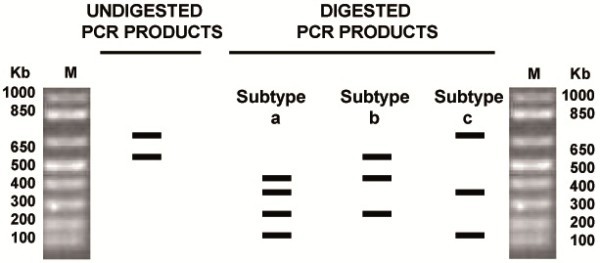
**Scheme expected restrictions patterns with*BstE *II of multiplex PCR product of BoHV-5 subtypes M: molecular weight marker.** The products of the undigested multiplex PCR showed two bands of 669 and 534 bp. The two bands, only the 669 bp one and only the 534 bp one are digested in subtypes **a**, **b** and **c** respectively.

**Figure 4:**
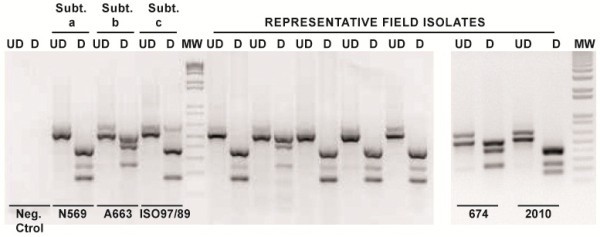
**Multiplex PCR-REA applied PCR amplicons before and after digestion with *BstE *II restrictions enzyme of the Argentine BoHV-5 representative isolates and reference strains.** The lanes corresponding to the most recent isolates are marked. Ud: undigested; D: digested; Subt: subtype; Neg.: Control Negative; Ref: reference strain. M: 1 Kb plus DNA size marker.

In the new assay developed in this work, the differential expected band pattern for each of the three BoHV-5 subtypes after multiplex PCR-REA is shown in Figure 3. The PCR products after digestion showed different cleaved combinations. The band of 669 bp is cleaved into two bands of 408 and 248 bp. Moreover the band of 534 bp is cleaved into two bands of 382 and 152 bp. Isolates 674 and 2010 showed band patterns similar to BoHV-5b and BoHV-5a prototypes, respectively (Figure 4). In addition, when DNA of the three different BoHV-1 subtypes was used as template, amplification products could not be digested by *BstE*II (data not shown).

### Sensitivity of the test

The multiplex PCR assay detected as few as 305 to 455 ng/μl of purified BoHV-5 DNA or approximately 203 to 303 genome copies. In the case of DNA extracted from infected tissue homogenates the assay detected down to 9.200 genome copies.

## Discussion

In countries like Argentina and Brazil where circulation of BoHV-5 is high and viral subtypes not described elsewhere in the world occur, there is a need for rapid and easy diagnostic tools which allow the classification of viral species and subtypes. In this work, a new multiplex PCR-REA that easily identifies all BoHV-5 subtypes was developed and improves previous assay reported by Claus [24]. The latter can be used as a confirmatory test for the detection of this virus and only allows species differentiation between BoHV-1 and BoHV-5, while our multiplex PCR – REA permits simultaneous subtyping of the isolates. This method was applied to 17 previously characterized virus isolates [11] and 2 newly identified ones. Results were identical to those obtained with the classical BoHV-5 subtyping technique (*BstE*II restriction profile of the entire genome). When different BoHV-1 subtypes were used as template for this multiplex PCR-REA, fragments of different molecular weight, as compared to BoHV-5, were obtained. Multiplex PCR-REA is more sensitive, faster, less laborious and more economical than the traditional whole genome restriction analysis, and can detect DNA both from purified virus and tissues.

The protocol uses two pairs of primers for the simultaneous amplification of UL27 and UL54 segments. Due to the molecular weight differences in the amplified fragments after digestion, it is possible to clearly distinguish the characteristic bands of each subtype by visual analysis of agarose gels. Amplicon sequencing allowed to find the point mutation responsible for the change on the restriction site of the enzyme *BstE*II and thus, for the different profiles obtained for a and c subtypes (G-1879 nt-A genbank accession number: NC_005261.2). This mutation was, until now, only observed in subtype c (ISO97/89) strain, isolated in Brazil [29]. We showed here the analysis of one out of two BoHV-5c subtypes reported until now in the world.

The method showed a detection sensitivity of 227 BoHV-5 genome copies of purified virus and 9.2 × 10^5^ BoHV-5 genome copies from tissue samples.

Nineteen BoHV-5 field isolates, including two newly identified ones, were subtyped using the described method. One of the two new isolates included in this work was isolated from cryopreserved bovine semen. To our knowledge, this is the first report of BoHV-5 virus identification from semen in Argentina. This sample was classified as subtype b by multiplex PCR-REA, constituting the last and one of the three BoHV-5b isolates characterized so far. Although no virulence differences between a and b subtypes have been found [30], subtype identification is relevant to understand virus genetic variability and contribute to molecular epidemiology studies. Noteworthy, the virally contaminated semen was harvested from an apparently healthy bull. This finding agrees with similar results obtained in Brazil and Australia [17,18]. Whether BoHV-5 transmitted to a cow via artificial insemination (AI) can cause neurological disorders in the recipient remains to be determined. Given the widespread use of AI to diversify cattle stocks, detection of animal viruses in semen, either by virus isolation or PCR, is crucial. In conclusion, the multiplex PCR-REA described in this work provides a new tool for the fast diagnosis and subtyping of BoHV-5. This development can aid in the understanding and control of these detrimental bovine viral infections.

## Conclusion

Fast and easy tools for the characterization of BoHV-5 viral isolates are required. Since, due to technical limitations, sequencing is not an option, the multiplex PCR – REA system described in this work provides an attractive tool for the improved control of BoHV-5 viral infections.

## Methods

### Cell culture and virus isolates

During a routine health test, a BoHV-5-positive semen sample was obtained in 2010 from a clinically healthy bovine from the Argentinean Province of Chaco (isolate 674). The semen sample was diluted 1:6 in fetal calf serum (FCS) and inoculated onto bovine testis cells in minimal essential medium (MEM) containing 10% FCS. Cells were incubated at 37°C in a 5% CO_2_ atmosphere, and daily checked for cytopathic effects (CPE). The second characterized isolate was obtained from an animal of about 4 months of age at weaning stage from the Argentine Province of Buenos Aires isolated in 2010 (isolate 2010). A typical outbreak with nervous symptoms and 20% lethality occurred in the 50-catle herd to which this calf belonged. One gram of brain tissue of this animal was homogenized, suspended in MEM containing 10% FCS and clarified at 11 000 × *g* for 20 min at 4°C. Then, half of the supernatant was inoculated into bovine testis cells in MEM containing 10% FCS and the other half was used for DNA extraction. Isolates 674 and 2010 originated therefore from samples sent to diagnostic laboratories for routine testing and are not subjected to a prior approval by the animal welfare committee. Three BoHV-5 reference strains were used as subtype controls: N569 (BoHV-5a), A663 (BoHV-5b) and ISO 97/87 (BoHV-5c). After standardization of the multiplex PCR assay, the method was tested on the two new isolates mentioned above and 17 previously characterized BoHV-5 (16 BoHV-5a and 1 BoHV-5b) field isolates [11].

### DNA extraction for PCR

The infected cell culture supernatant from semen and the supernatant of homogenized tissue of the BoHV-5 positive sample were subjected to DNA extraction using QIAamp DNA Mini kit (Qiagen, Hilden, Germany) according to the manufacturer’s protocol. Purified DNA was stored at - 20°C until testing. DNA concentration was deduced from absorbance measured in a spectrophotometer.

### Differential PCR for BoHV-1 and BoHV-5

To identify the viral species of isolates 674 and 2010, the multiplex PCR designed by Claus and collaborators [24] was carried out. Amplification products were of 354 bp and 159 bp for BoHV-1 and BoHV-5, respectively. Products were analyzed on 1% agarose gel electrophoresis, stained with ethidium bromide (0.5 μg/ml) in TBE buffer (89 mM Tris, 89 mM boric acid, 2 mM EDTA, pH8.4), and visualized under UV light.

### Classic subtyping of BoHV-5 by restriction endonuclease analysis (REA)

Field isolates 674 and 2010 were inoculated in tissue culture flaks (175 cm^2^) with nearly confluent, overnight grown MDBK (Madin Darby bovine kidney) monolayers, at a multiplicity of infection of 0.1, and incubated at 37°C and 5% CO_2_. Post infection cultures were frozen at −80°C. After two successive rounds of freezing and thawing, clarification was carried out at 3000 rpm for 20 min at 4°C. Purification and extraction of viral DNA was performed as detailed by Maidana et al. [11].

Four μg of viral DNA from each reference strain and field isolate (2010 and 674) were incubated overnight with *BstE*II restriction enzyme (1U) under the conditions recommended by the manufacturer (Promega, Wisconsin, USA). Digestion products were separated overnight by electrophoresis on 0.7% agarose gels at 50 V using TBE buffer. Gels were stained with ethidium bromide and photographed under UV light.

### Identification of differential restriction sites between subtype a and c

Although differential PCRs to discriminate between BoHV-1 and BoHV-5, or between BoHV5a and b subtypes have been developed [11,24], subtype c is still indistinguishable by these techniques. *In silico* analysis showed that a site included in the open reading frame of the UL54 gene serves to differentiate subtype c from the other two. Primers were designed based on the published sequence of BoHV-5 (Genbank accession number: NC_005261.2) (UL54F: TAT-AAC-CCC-CTC-AAC-AAA-AT (nt 1631 to 1650) and UL54R: TCT-GCG-AGT-ACC-AGG-TGC-CG nt 2280 to 2300). DNA sequence analysis to locate polymorphic regions within the UL54 target gene was performed using Vector NTI Suite version 8.0 (Invitrogen, Merelbeke, Belgium).

### The assay was performed with purified DNA from strains of different subtypes

Amplification was carried out in a 50 μl reaction mix containing 5 ng of template DNA, Taq DNA polymerase buffer (NEB, Ipswich, MA, USA), 2 mM MgCl_2_, 6% DMSO, 200 μM dNTPs, 0.3 μM of both forward and reverse primers and 1U Taq DNA polymerase (NEB). Annealing temperatures were optimized for each primer pair. The PCR program consisted of 10 min at 96°C, followed by 35 cycles of 1 min at 96°C; 1 min at 58°C and 1 min at 72°C with a final extension step of 10 min at 72°C.The resulting products were separated by electrophoresis in 1% agarose gels and visualized under UV light after ethidium bromide staining. Amplified products were purified using Illustra GFX™ PCR, DNA and gel band purification kit (GE Healthcare, Diegem, Belgium). The quality of all DNA preparations was evaluated by agarose gel electrophoresis. Sequencing reactions were performed with BigDye Terminator v3.0 kit (Applied Biosystems, Lennik, Belgium) and analyzed in an ABI Prism 3730 DNA Analyzer (Applied Biosystems).

Each PCR product of reference strains was sequenced twice in both directions using forward and reverse primers. Nucleotide and predicted amino acid sequences were edited; aligned and analyzed with BioEdit version 7.0.5.3 [31] to determine single nucleotide polymorphisms at *BstE*II restriction sites.

### Multiplex PCR-REA assay for BoHV-5 subtyping

UL27 PCR assay [11] was modified for easy discrimination between three BoHV-5 subtypes. The above described UL54 primers that amplify a fragment of 669 bp were incorporated into the UL27 PCR [11]. As template, total genomic material of the three subtypes of BoHV-5 was used. The expected results of this multiplex PCR are two bands of 669 bp and 534 bp, respectively. The reaction mix with two pair primers (UL54F-UL54R, UL27F-UL27R) and the PCR program used were the same as described above.

Aliquots (25 μl) of the PCR products were incubated with *BstE*II, while the remaining 25 μl were used as undigested control. The resulting products were separated by electrophoresis in 1% agarose gels and visualized under UV light after ethidium bromide staining. The pattern of expected products before and after digestion is shown in Figure 3.

The applicability of multiplex PCR-REA was demonstrated on reference strains corresponding to the 3 different subtypes of each BoHV-5 as well as on 17 previously characterized BoHV-5 fields isolates [11].

### Sensitivity of the test

To evaluate the sensitivity of the multiplex PCR, 10-fold serial dilutions of DNA obtained from different types of samples (purified virus and infected tissue homogenates) were used as template. Then copy numbers were calculated based on mass estimations of the entire genome of BoHV-5 mass and of the DNA samples analyzed.

## Competing interests

The authors declare that there are no competing interests.

## Authors’ contributions

SM and SR designed the experiments, analyzed the data and drafted the manuscript together. SM performed the experiments. FC, PR and BS kindly provided the reference strains. ET and AM participated in the interpretation of data and preparation of the manuscript draft. CM, DC and GD helped with virus amplification on cell cultures. All authors read and approved the final manuscript.
